# Supplementary material for: The SMC5/6 Complex Represses the Replicative Program of High-Risk Human Papillomavirus Type 31
Source: Pathogens. 2020 Sep 25;9(10):786. doi: 10.3390/pathogens9100786 (PMC7599729; doi:10.3390/pathogens9100786)
Supplement: Supplementary file 1 [file pathogens-09-00786-s001.pdf]

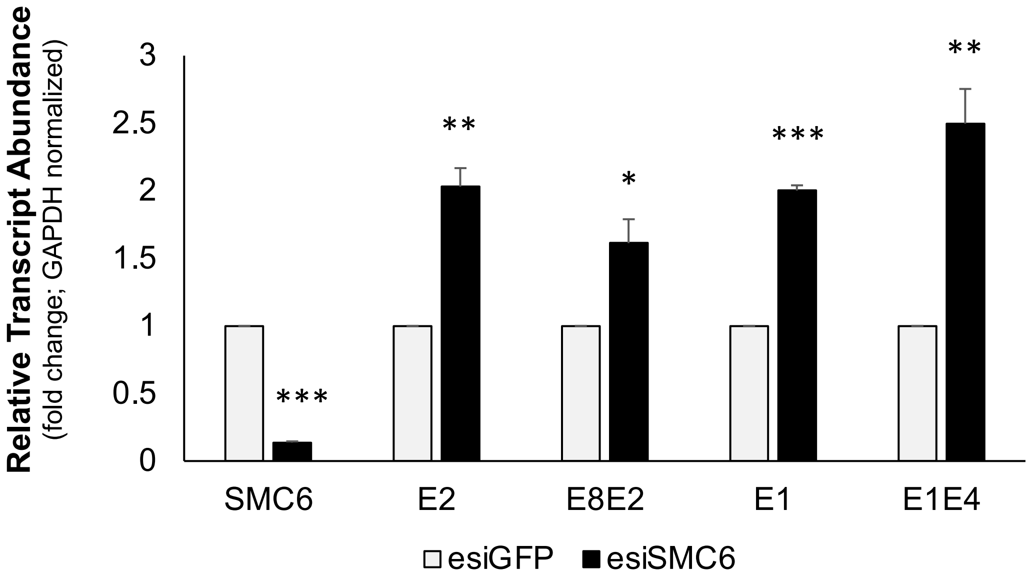

Supplementary Figure 1. SMC6 knockdown increases HPV-31 transcript abundance in CIN612-9E cells. Forty-eight hour siRNA knockdown of SMC6 resulted in decreased SMC6 and increased HPV-31 E2, E8E2, E1 and E1E4 transcripts in CIN612-9E cells. C_t values were normalized to GAPDH and fold change shown relative to control (set to 1; n=3). The experiments was performed 3 independent times and statistical significance determined by two-tailed Student's T-test where * = $p \leq 0.05$, ** = $p \leq 0.005$ and *** $p = \leq 0.0005$.
